# Enthalpy–Entropy Trade-Off Underlies Geometric Isomer Selectivity in Histamine H_1_ Receptor–Doxepin Interaction

**DOI:** 10.1021/acsmedchemlett.5c00696

**Published:** 2026-01-27

**Authors:** Hiroto Kaneko, Satoru Nagatoishi, Kouhei Tsumoto, Tadashi Ando, Mitsunori Shiroishi

**Affiliations:** † Department of Biological Science and Technology, 378550 Tokyo University of Science, 6-3-1 Niijuku, Katsushika-ku, Tokyo 125-8585, Japan; ‡ Department of Bioengineering, School of Engineering, The University of Tokyo, 7-3-1 Hongo, Bunkyo-ku, Tokyo 113-8656, Japan; § Department of Applied Electronics, Tokyo University of Science, 6-3-1 Niijuku, Katsushika-ku, Tokyo 125-8585, Japan; ∥ Research Institute for Science and Technology, Tokyo University of Science, 2641 Yamazaki, Noda, Chiba 278-8510, Japan

**Keywords:** Histamine H_1_ receptor, Isothermal titration calorimetry, Enthalpy−entropy compensation, Doxepin isomers, Molecular dynamics simulation

## Abstract

Understanding the thermodynamic basis of ligand recognition by G-protein-coupled receptors (GPCRs) is crucial for rational drug design. Here, we directly characterized the binding thermodynamics of the histamine H_1_ receptor (H_1_R) interacting with the geometric isomers of doxepin using isothermal titration calorimetry combined with molecular dynamics (MD) simulations. The *Z*-isomer binding to H_1_R_WT exhibited a larger enthalpic gain but a greater entropic loss than the *E*-isomer, whereas these differences were diminished in the T112^3.37^V mutant. Cluster analysis of MD trajectories revealed that *Z*-doxepin adopts a more restricted conformation upon binding, consistent with its enthalpy-driven interaction and reduced conformational entropy. These findings indicate that H_1_R distinguishes between *E*- and *Z*-isomers not only by affinity but also through distinct thermodynamic fingerprints. This study provides mechanistic insight into the enthalpy–entropy trade-off in GPCR–ligand interactions, highlighting the importance of conformational restriction and flexibility in designing ligands with optimized thermodynamic and functional properties.

## Graphical abstract

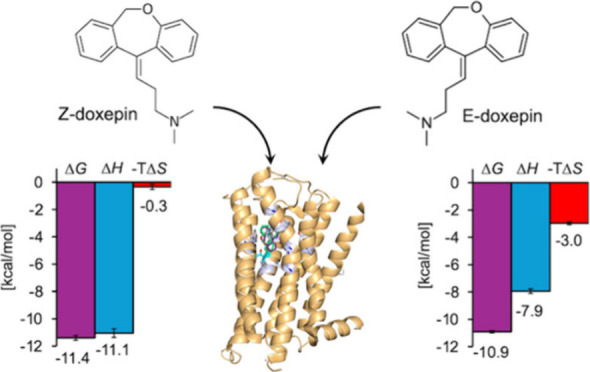

G protein–coupled receptors (GPCRs) constitute a large family of membrane proteins that recognize hormones, neurotransmitters, and drugs, thereby regulating a wide variety of signaling pathways,^1^ and more than 30% of currently marketed drugs target GPCRs.^2^ Among them, the histamine H_1_ receptor (H_1_R) is a subtype involved in allergic and inflammatory responses, vascular permeability, airway constriction, and regulation of wakefulness and cognitive functions in the central nervous system.^3^ H_1_R has long been a major pharmacological target of antihistamines. First-generation antihistamines exhibit low receptor selectivity and cause central side effects, such as sedation, whereas second-generation drugs have improved selectivity but sometimes limited therapeutic efficacy. These limitations highlight the clinical and scientific importance of exploring H_1_R ligands from new perspectives.^4^ Doxepin, a tricyclic antidepressant that also acts as a potent H_1_R antagonist, exists as two geometric (*E*/*Z*) isomers differing in the configuration around the central double bond (Figure 1a). We previously determined the crystal structure of the H_1_R–doxepin complex, providing a structural framework for understanding H_1_R–antihistamine recognition^5^ (Figure 1b). In a separate study, we analyzed the binding of doxepin to the purified H_1_R using a racemic mixture of its *E*- and *Z*-isomers and demonstrated that the *Z*-isomer exhibits approximately 5-fold higher affinity than the *E*-isomer.^6^ Furthermore, we identified Thr112^3.37^ as a key residue contributing to this isomer-dependent selectivity.

**Figure 1:**
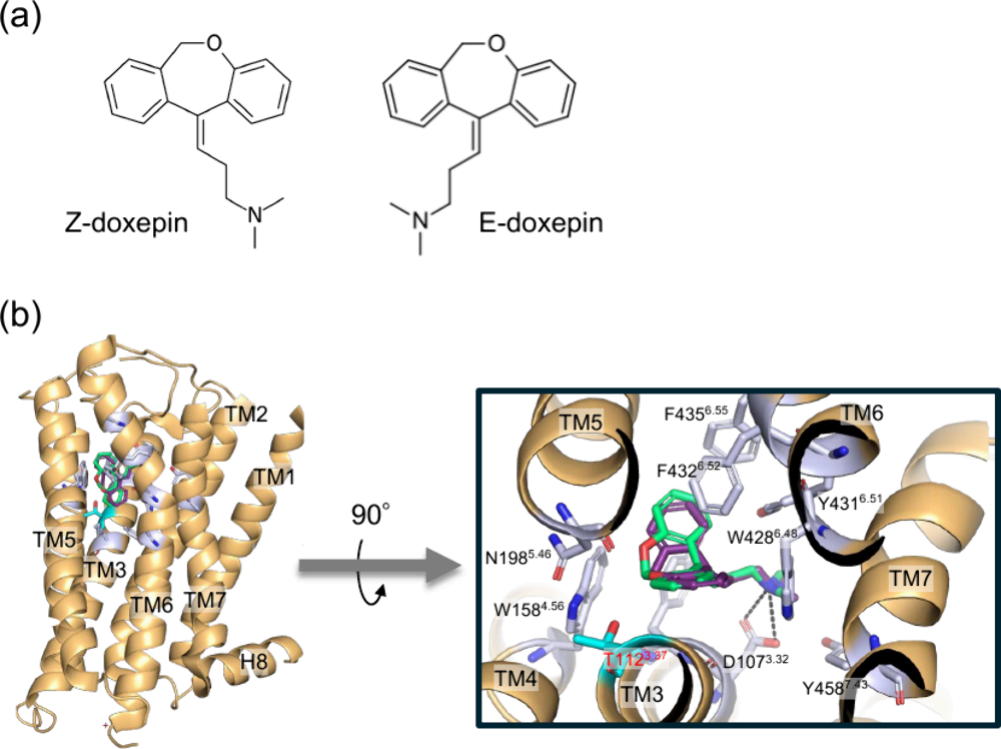
**Structure of the H**_**1**_ **R–doxepin complex.** (a) Chemical structures of the doxepin isomers. (b) Crystal structure of the H_1_R–doxepin complex (PDB ID: 3RZE). The backbone of H_1_R is shown in khaki. The *E*-doxepin and *Z*-doxepin molecules are represented as purple and spring-green sticks, respectively. H_1_R residues interacting with doxepin are depicted as gray sticks.

In recent rational drug design, thermodynamic and kinetic information regarding the interactions between target proteins and ligands has proven to be highly valuable beyond simply binding affinity. Rather than focusing solely on the binding affinity (*K*_d_) or the Gibbs free energy change (Δ*G*), elucidating the respective contributions of enthalpy (Δ*H*) and entropy (−*T*Δ*S*) provides important guidance for improving the quality and selectivity of hit compounds.^7,8^ In particular, enthalpy–entropy compensation has attracted attention as a key concept for explaining ligand selectivity and isomer specificity. However, the molecular origins of this compensation are diverse and remain the subject of ongoing debate, as they can arise from various factors such as changes in solvation, conformational flexibility, and intermolecular interactions.^9,10^ One of the pioneering studies of thermodynamic analyses of GPCRs was conducted by Borea and colleagues in the 1990s. They performed radioligand binding assays at multiple temperatures and derived the Δ*H* and Δ*S* of ligand binding from van’t Hoff plots. Using this approach, they discussed the thermodynamic parameters of agonists and antagonists for the A1 and A2 adenosine receptors as well as the CB1 and CB2 cannabinoid receptors.^11–13^ Similar van’t Hoff analyses have also been conducted for H_1_R, revealing the overall thermodynamic characteristics of ligand binding and the role of Lys179^ECL2^ and Lys191^5.39^.^14,15^ However, the structural origins of these thermodynamic parameters, namely, how specific molecular interactions within the receptor–ligand complex give rise to the observed enthalpic and entropic contributions, remain largely unexplored.

Isothermal titration calorimetry (ITC) is a powerful technique for studying the thermodynamics of protein–ligand interactions, as it enables direct and quantitative analysis of binding events and allows simultaneous determination of *K*_d_, Δ*H*, and Δ*S*. However, its application to membrane proteins remains a significant challenge due to experimental limitations such as sample instability and the difficulty of achieving sufficiently high yields of proteins.^16^ Consequently, reports of direct ITC measurements of GPCR–ligand interactions are extremely limited, and to our knowledge they are restricted to studies of CCR5 chemokine receptor with its agonist RANTES,^17^ and the β_2_-adrenergic receptor with a negative allosteric modulator.^18^ Nevertheless, these studies did not provide detailed discussions of the thermodynamic parameters involved.

In our previous study, we evaluated the binding affinities of the *E*- and *Z*-isomers of doxepin to purified H_1_R by analyzing the ratio of receptor-bound isomers using HPLC.^6^ The results indicated that the *Z*-isomer exhibited approximately 5-fold higher affinity for H_1_R than the *E*-isomer. In contrast, the T112^3.37^V mutant showed comparable affinities for both isomers, suggesting that Thr112^3.37^ is involved in the isomer selectivity of doxepin binding. Although the oxygen atom of doxepin is located within a distance suitable for hydrogen bonding with the hydroxyl group of Thr112^3.37^, our MD simulations revealed that such geometries are rarely observed, implying that hydrogen bonding is unlikely to be the major factor responsible for the observed selectivity. Because the structural and energetic basis underlying the higher affinity of the *Z*-isomer remains unclear, we then performed a detailed thermodynamic analysis of the H_1_R–doxepin interaction using ITC to characterize the energetic and molecular determinants of the isomer-dependent binding.

In this study, the construct referred to as H_1_R_WT corresponds to the one used in our previous crystallographic analysis,^5^ in which the N-terminal 19 residues were deleted and T4 lysozyme was fused into the third intracellular loop. The T112^3^·^37^V mutant was generated by introducing a point mutation into this construct. We expressed these proteins in large quantities using the *Saccharomyces cerevisiae* expression system and purified them by TALON resin affinity chromatography followed by size-exclusion chromatography as the final purification step (Supplementary Figure 1). We first performed thermodynamic analyses using ITC to characterize the interactions between doxepin (E/Z mixture) and H_1_R_WT or the T112^3.37^V mutant, respectively (Supplementary Figure 2). The obtained thermodynamic parameters (Δ*G*, Δ*H*, and – *T*Δ*S*) are summarized in Table 1. Although no substantial difference in binding affinity was observed between the wild-type and mutant receptors, their enthalpic and entropic contributions differed. The interaction between H_1_R_WT and doxepin was characterized as enthalpy–entropy driven, with a dominant enthalpic contribution and a relatively small entropic component. (Figure 2a). This enthalpic contribution likely arises mainly from van der Waals and other nonpolar interactions rather than hydrogen bonding, as our previous study demonstrated that Thr112^3.37^ and the oxygen atom of doxepin do not form a hydrogen bond.^6^ In contrast, the T112^3.37^V mutant exhibited a smaller binding enthalpy but a greater entropic contribution (Figure 2b). These results suggest that Thr112^3.37^ contributes to enthalpic stabilization in doxepin recognition by H_1_R.

**Figure 2:**
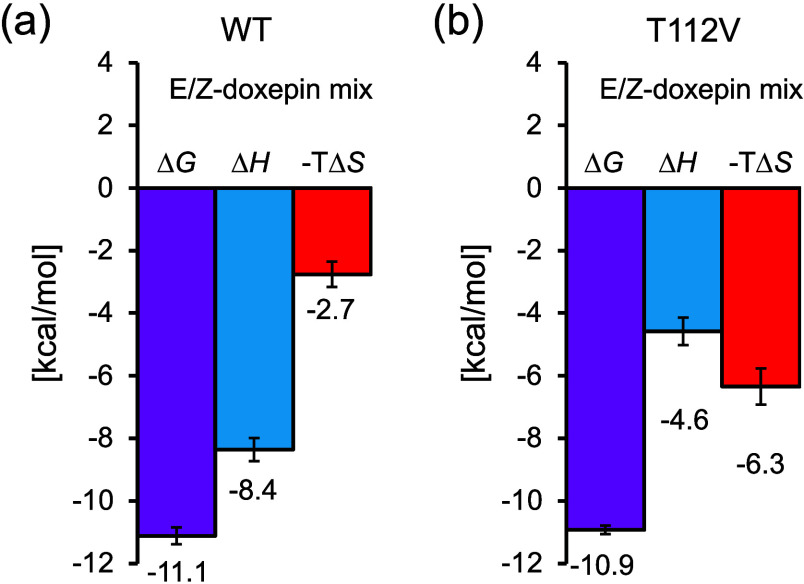
**Thermodynamic profiles of the interactions between H**_**1**_ **R and doxepin (*E*/*Z* mixture).** (a) Interaction between H_1_R_WT and doxepin (*E*/*Z* mixture). (b) Interaction between H_1_R_T112^3.37^V and doxepin (*E*/*Z* mixture). The graphs were generated based on the thermodynamic parameters summarized in Table 1.

**Table 1:** Thermodynamic Parameters of H_1_R_WT and T112^3.37^V Binding to Doxepin (*E*/*Z* Mixture and Individual Isomers) Determined by ITC^a^

	*N*	*K*_d_ [nM]	Δ*H* [kcal/mol]	–*T*Δ*S* [kcal/mol]	Δ*G* [kcal/mol]
WT–Mix	0.57 ± 0.06	8.0 ± 3.0	–8.4 ± 0.5	–2.7 ± 0.6	–11.1 ± 0.2
WT–*E*	0.56 ± 0.02	10.0 ± 1.3	–7.9 ± 0.2	–3.0 ± 0.1	–10.9 ± 0.1
WT–*Z*	0.58 ± 0.01	4.5 ± 1.4	–11.1 ± 0.3	–0.3 ± 0.2	–11.4 ± 0.2
T112^3.37^V–Mix	0.72 ± 0.08	9.8 ± 2.2	–4.6 ± 0.4	–6.3 ± 0.6	–10.9 ± 0.1
T112^3.37^V–*E*	0.72 ± 0.05	6.2 ± 2.7	–4.2 ± 0.2	–7.0 ± 0.5	–11.2 ± 0.3
T112^3.37^V–*Z*	0.75 ± 0.04	15.5 ± 12.4	–4.2 ± 0.1	–6.7 ± 0.7	–10.9 ± 0.7

a Mix denotes doxepin (*E*/*Z* mixture), and *N* represents the stoichiometry. The data are expressed as the mean ± standard deviation (SD) from three independent measurements. However, for the H_1_R_T112^3.37^V–doxepin (*E*/*Z* mixture) interaction, the values represent the mean ± SD from two independent measurements. As described in the Experimental Procedures, the concentrations of each isomer were corrected and the ITC data were reanalyzed accordingly.

Next, we obtained the individual *E*- and *Z*-isomers of doxepin and performed thermodynamic analyses of their interactions with H_1_R_WT and the T112^3.37^V mutant. The corresponding thermograms and reanalyzed binding curves are shown in Supplementary Figure 3. Initially, the concentrations of each isomer solution were determined by matching the absorbance at 280 nm to that of doxepin (*E*/*Z* mixture). However, the stoichiometries (*N* values) obtained from the ITC measurements differed between the *E*- and *Z*-isomers, even though the same protein preparation was used, suggesting that the ligand concentrations were inaccurate. Therefore, the concentrations were corrected based on the absorbance at 254 nm, which is used for the quantitative analysis of doxepin in the U.S. Pharmacopeia (USP),^19^ and the ITC data were reanalyzed (details are provided in Experimental Procedures). The resulting thermodynamic parameters (Δ*G*, Δ*H*, and – *T*Δ*S*) are summarized in Figure 3 and Table 1. The interactions between H_1_R_WT and doxepin isomers were overall enthalpy-driven (Figure 3a). When the two isomers were compared, Z-doxepin showed a 3.2 kcal/mol larger binding enthalpy gain but a 2.6 kcal/mol greater entropic penalty than *E*-doxepin. In contrast, for the H_1_R_T112^3.37^V mutant, both isomers exhibited similar values of binding enthalpy and entropy (Figure 3b). In our previous study, we reported that the *Z*-isomer displayed approximately 5.2-fold higher affinity for wild-type H_1_R compared with the *E*-isomer, whereas no difference in affinity was observed for the T112^3.37^V mutant. Consistent with those findings, the ITC analysis in this study also showed that the *Z*-isomer tended to bind with higher affinity to the wild-type receptor, while the two isomers exhibited comparable affinities for the mutant receptor. Taken together, these findings indicate that the hydroxyl group of Thr112^3.37^ contributes to the balance between enthalpic gain and entropic loss during ligand binding, with a more pronounced effect in the interaction with the *Z*-isomer, thereby shaping the observed isomer selectivity.

**Figure 3:**
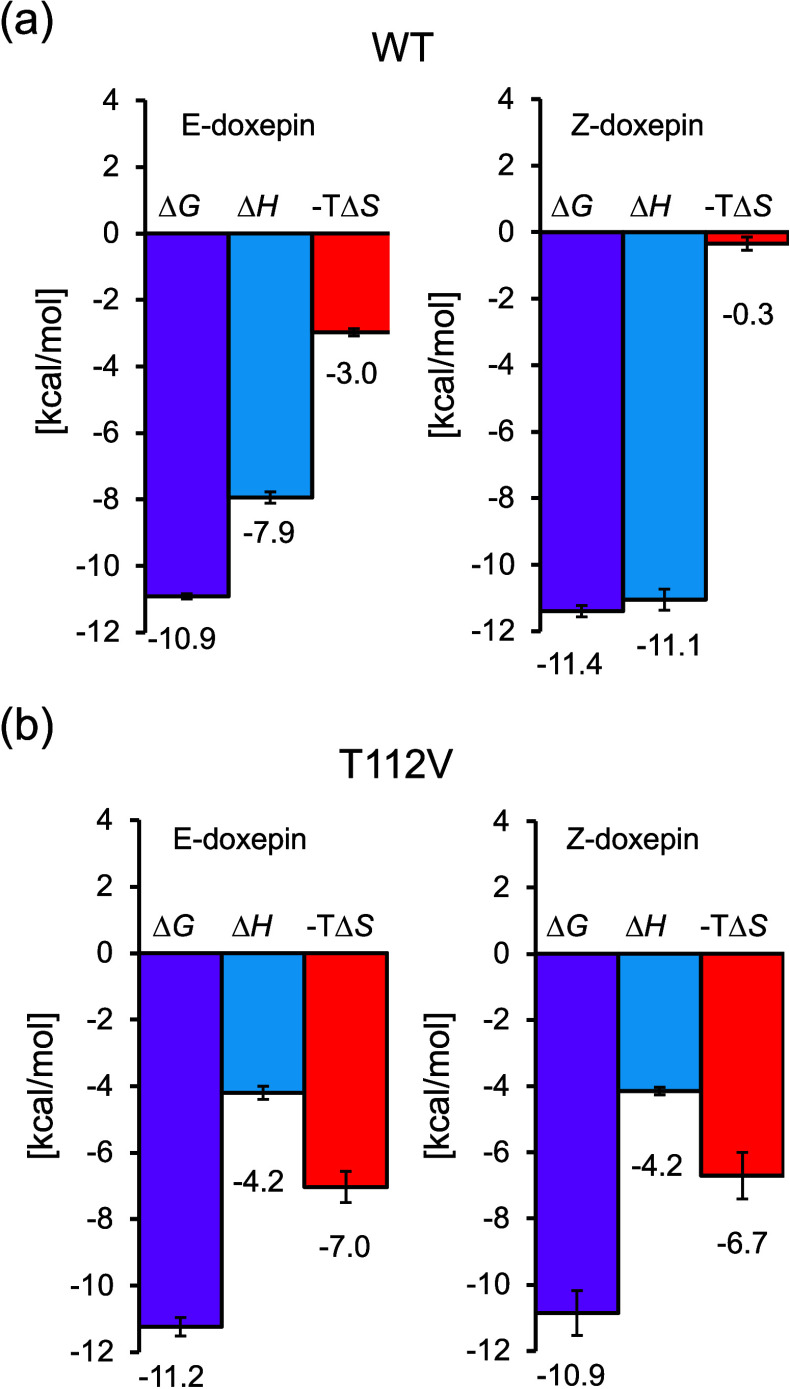
**Thermodynamic profiles of the interactions between H**_**1**_ **R and doxepin isomers.** (a) Interactions of H_1_R_WT with *E*-doxepin and *Z*-doxepin. (b) Interactions of H_1_R_T112^3.37^V with *E*-doxepin and *Z*-doxepin. The graphs were generated based on the thermodynamic parameters summarized in Table 1.

ITC measurements revealed distinct thermodynamic profiles for the interactions between H_1_R and doxepin isomers. In particular, the binding of the *Z*-isomer to the H_1_R_WT was characterized by a larger entropic penalty compared with that of the *E*-isomer, whereas the thermodynamic differences between the isomers were largely diminished in the T112^3.37^V mutant. Our previous studies using MD simulations of the H_1_R-doxepin complexes revealed no significant structural differences within or around the H_1_R orthosteric pocket between the two doxepin isomers, and no water molecules were observed near Thr112^3.37^ or Val112^3.37^ in the ligand-bound states.^6^ These findings indicate that the thermodynamic differences observed between the WT and T112^3.37^V mutant receptors in this study are unlikely to arise from variations in direct doxepin–residue interactions, hydration states, or receptor conformational changes but rather arise from differences in the reduction of doxepin’s conformational entropy upon binding.

To examine this possibility, we performed a cluster analysis of doxepin conformations before and after receptor binding using MD simulation data (Figure 4). Prior to binding, both isomers displayed similar cluster distributions (Figure 4a). However, upon binding to H_1_R_WT, the *Z*-isomer exhibited a pronounced bias toward several specific clusters (Clusters 1–10), whereas the *E*-isomer retained a broader conformational distribution (Figure 4b). In contrast, when bound to the T112^3.37^V mutant, both isomers showed conformational distributions similar to those of the unbound state (Figure 4c). These results suggest that in the WT receptor the hydroxyl group of Thr112^3.37^ may allow the *Z*-isomer to fit more snugly into the binding pocket and stabilize a specific conformation, thereby gaining greater enthalpic benefit at the cost of conformational entropy. This interpretation is consistent with the ITC data, which revealed that the binding of the *Z*-isomer to H_1_R_WT was associated with a larger enthalpic gain but a greater entropic loss than the *E*-isomer. In contrast, in the T112^3.37^V mutant, both isomers appear to retain higher conformational flexibility upon binding, resulting in similar thermodynamic profiles dominated by a larger entropic contribution. Such an enthalpy–entropy compensation driven by ligand conformational entropy has also been demonstrated in the interaction between galectin-3 and its stereoisomeric ligands through a combination of ITC, X-ray crystallography, NMR, and MD simulations.^20^ Collectively, these observations highlight a mechanistic link between the thermodynamic signature obtained from ITC and the conformational behavior observed in MD simulations, suggesting that the enthalpy–entropy trade-off underlying the isomer selectivity of doxepin can be largely attributed to the conformational restriction of the *Z*-isomer upon binding.

**Figure 4:**
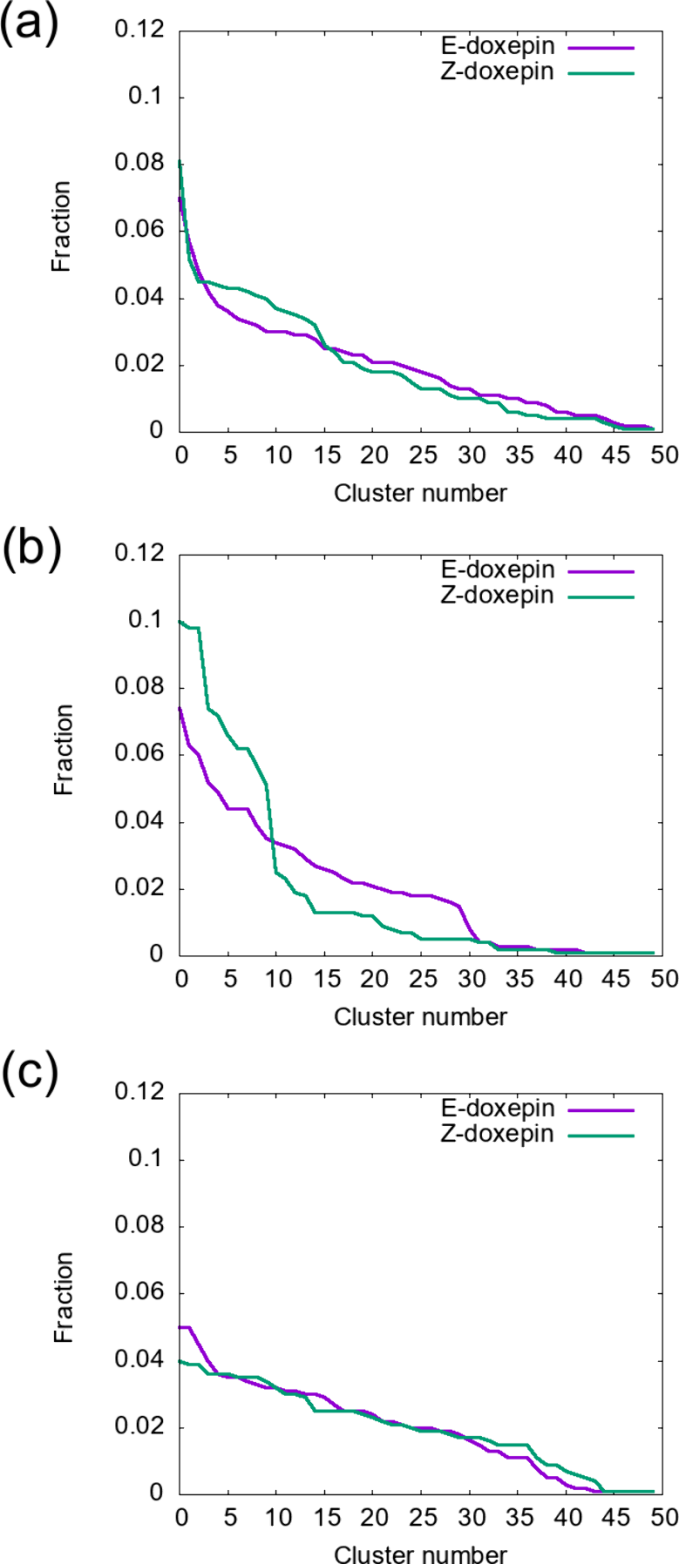
**Fractional distribution of doxepin conformational clusters.** (a) Free doxepin in solution, (b) doxepin bound to H_1_R_WT, and (c) doxepin bound to the T112^3.37^V mutant H_1_R. The *k*-means algorithm with *k* = 50 was used to cluster the conformations.

This study directly characterized the thermodynamic profiles of the histamine H_1_ receptor (H_1_R) interacting with the geometric isomers of doxepin using ITC. The *Z*-Isomer binding to H_1_R_WT exhibited a larger enthalpic gain but a greater entropic loss than the *E*-isomer, whereas these differences were diminished in the T112^3.37^V mutant. MD cluster analysis revealed that the *Z*-isomer adopts a more restricted conformation upon binding, which is consistent with the observed enthalpy–entropy trade-off. Together, these findings demonstrate that H_1_R discriminates between the *E*- and *Z*-isomers not only by affinity but also through distinct thermodynamic fingerprints. While the present analysis suggests that differences in ligand conformational restriction contribute to the observed enthalpy–entropy compensation, contributions from solvent reorganization and hydration effects cannot be completely excluded. However, both the crystal structure and our MD simulations showed no persistent water molecules at the ligand–receptor interface for either isomer, implying that solvent-related contributions are likely comparable between the E- and Z-isomers under the experimental conditions used in this study. Accordingly, the observed thermodynamic differences are most consistently interpreted as arising from differences in the ligand conformational entropy rather than major changes in receptor hydration. Future work employing a reconstituted membrane system such as nanodiscs could therefore provide a more physiologically relevant view of GPCR–ligand energetics. Extending this combined thermodynamic and computational approach to other GPCRs, including agonist–antagonist pairs and biased ligands, will deepen our understanding of how environmental context, receptor flexibility, and ligand chemistry collectively define enthalpy–entropy balance and functional selectivity. Ultimately, such insights will aid rational drug design by enabling the development of ligands optimized not only for affinity but also for thermodynamic and conformational efficiency.

## Supplementary Material



## Notes

Experimental procedures, purification profiles of H_1_R by size-exclusion chromatography, ITC thermograms, and UV–visible absorption spectra of doxepin (PDF)

## Abbreviations

GPCR: G protein-coupled receptor
ITC: isothermal titration calorimetry
MD: molecular dynamics
WT: wild-type
HPLC: high performance liquid chromatography
TEV: tobacco etch virus
T4L: T4 lysozyme
GFP: green fluorescent protein
DMSO: dimethyl sulfoxide
DDM: n-dodecyl-β-d-maltoside
CHS: cholesteryl hemisuccinate
SDS-PAGE: sodium dodecyl sulfate-polyacrylamide gel electrophoresis

## References

[1] Zhang M., Chen T., Lu X., Lan X., Chen Z., Lu S. (2024). G protein-coupled receptors (GPCRs): advances in structures, mechanisms,
and drug discovery. Signal Transduct Target
Ther.

[2] Lorente J. S., Sokolov A. V., Ferguson G., Schioth H. B., Hauser A. S., Gloriam D. E. (2025). GPCR drug discovery:
new agents, targets and indications. Nat. Rev.
Drug Discov.

[3] Simons F. E. (2004). Advances
in H1-antihistamines. N Engl J. Med..

[4] Simon F. E., Simons K. J. (2008). H1 antihistamines:
current status and future directions. World
Allergy Organ J..

[5] Shimamura T., Shiroishi M., Weyand S., Tsujimoto H., Winter G., Katritch V., Abagyan R., Cherezov V., Liu W., Han G. W., Kobayashi T., Stevens R. C., Iwata S. (2011). Structure
of the human histamine H1 receptor complex with doxepin. Nature.

[6] Kaneko H., Korenaga R., Nakamura R., Kawai S., Ando T., Shiroishi M. (2024). Binding characteristics of the doxepin E/Z-isomers
to the histamine H(1) receptor revealed by receptor-bound ligand analysis
and molecular dynamics study. J. Mol. Recognit.

[7] Ferenczy G. G., Keseru G. M. (2020). Thermodynamic profiling
for fragment-based lead discovery
and optimization. Expert Opin Drug Discov.

[8] Geschwindner S., Ulander J., Johansson P. (2015). Ligand Binding
Thermodynamics in
Drug Discovery: Still a Hot Tip?. J. Med. Chem..

[9] Dragan A. I., Read C. M., Crane-Robinson C. (2017). Enthalpy-entropy
compensation: the
role of solvation. Eur. Biophys J..

[10] Peccati F., Jimenez-Oses G. (2021). Enthalpy-Entropy
Compensation in Biomolecular Recognition:
A Computational Perspective. ACS Omega.

[11] Borea P. A., Dalpiaz A., Varani K., Guerra L., Gilli G. (1995). Binding thermodynamics
of adenosine A2a receptor ligands. Biochem.
Pharmacol..

[12] Dalpiaz A., Gessi S., Borea P. A., Gilli G. (1995). Binding thermodynamics
of serotonin to rat-brain 5-HT1A, 5HT2A and 5-HT3 receptors. Life Sci..

[13] Merighi S., Simioni C., Gessi S., Varani K., Borea P. A. (2010). Binding
thermodynamics at the human cannabinoid CB1 and CB2 receptors. Biochem. Pharmacol..

[14] Wittmann H. J., Seifert R., Strasser A. (2009). Contribution of binding
enthalpy
and entropy to affinity of antagonist and agonist binding at human
and guinea pig histamine H(1)-receptor. Mol.
Pharmacol..

[15] Kobayashi C., Tanaka A., Yasuda T., Hishinuma S. (2020). Roles of Lys191
and Lys179 in regulating thermodynamic binding forces of ligands to
determine their binding affinity for human histamine H(1) receptors. Biochem. Pharmacol..

[16] Rajarathnam K., Rosgen J. (2014). Isothermal titration calorimetry
of membrane proteins
- progress and challenges. Biochim. Biophys.
Acta.

[17] Nisius L., Rogowski M., Vangelista L., Grzesiek S. (2008). Large-scale expression
and purification of the major HIV-1 coreceptor CCR5 and characterization
of its interaction with RANTES. Protein Expr
Purif.

[18] Ahn S., Kahsai A. W., Pani B., Wang Q. T., Zhao S., Wall A. L., Strachan R. T., Staus D. P., Wingler L. M., Sun L. D., Sinnaeve J., Choi M., Cho T., Xu T. T., Hansen G. M., Burnett M. B., Lamerdin J. E., Bassoni D. L., Gavino B. J., Husemoen G., Olsen E. K., Franch T., Costanzi S., Chen X., Lefkowitz R. J. (2017). Allosteric
″beta-blocker″ isolated from a DNA-encoded small molecule
library. Proc. Natl. Acad. Sci. U. S. A..

[19] Monograph: Doxepin Hydrochloride. In USP-NF; USP: Rockville, MD, 2018. https://www.uspnf.com/sites/default/files/usp_pdf/EN/USPNF/errata467DoxepinHydrochloride.pdf.

[20] Verteramo M. L., Stenstrom O., Ignjatovic M. M., Caldararu O., Olsson M. A., Manzoni F., Leffler H., Oksanen E., Logan D. T., Nilsson U. J., Ryde U., Akke M. (2019). Interplay
between Conformational Entropy and Solvation Entropy in Protein-Ligand
Binding. J. Am. Chem. Soc..

