# Supplementary material for: Enthalpy–Entropy Trade-Off Underlies Geometric Isomer Selectivity in Histamine H1 Receptor–Doxepin Interaction
Source: ACS Med Chem Lett. 2026 Jan 27;17(2):490–4. doi: 10.1021/acsmedchemlett.5c00696 (PMC12907940; doi:10.1021/acsmedchemlett.5c00696)
Supplement: Supplementary file 1 [file ml5c00696_si_001.pdf]

Supporting Information

Enthalpy–entropy trade-off underlies geometric isomer selectivity in histamine H_1 receptor–doxepin interaction

Hiroto Kaneko[1], Satoru Nagatoishi[2], Kouhei Tsumoto[2], Tadashi Ando*[3,4], Mitsunori Shiroishi*[1,4]

1 Department of Biological Science and Technology, Tokyo University of Science, 6-3-1 Niijuku,
2 Department of Bioengineering, School of Engineering, The University of Tokyo, 7-3-1 Hongo, Bunkyo-ku, Tokyo 113-8656, JAPAN
3 Katsushika-ku, Tokyo, 125-8585, JAPAN
Department of Applied Electronics, Tokyo University of Science, 6-3-1 Niijuku, Katsushika-ku, Tokyo, 125-8585, JAPAN
4 Research Institute for Science and Technology, Tokyo University of Science, 2641 Yamazaki, Noda, Chiba 278-8510, JAPAN

Experimental Procedures
Supplementary Figure 1
Supplementary Figure 2
Supplementary Figure 3
Supplementary Figure 4

Experimental Procedures

Preparation of H_1R_WT and $H_1R\_T112^{3.37}V$

H_1R_WT and $H_1R\_T112^{3.37}V$ plasmids used in this study were the same constructs described previously [1]. The wild-type construct corresponds to the H_1R-T4L variant originally developed for crystallographic analysis, in which T4 lysozyme was inserted into the third intracellular loop (Gln222–Gly404) and 19 residues were truncated from the N-terminus. This gene was cloned into the Saccharomyces cerevisiae expression vector pDDGFP-2 under the control of the GAL1 promoter, resulting in fusion with GFP and a C-terminal His8-tag via a TEV protease site. The $T112^{3.37}V$ mutant plasmid was generated previously by site-directed mutagenesis of this WT construct. For protein production, the yeast strain FGY217 (MATα, ura3-52, lys2Δ201, pep4Δ) was transformed with the plasmids using the lithium acetate method, as described earlier [2]. Colonies were selected on uracil-deficient medium, expanded in liquid culture, and transferred to nutrient-rich galactose-containing medium supplemented with DMSO to induce receptor expression under the GAL1 promoter. Cells were harvested by centrifugation, disrupted with glass beads in breaking buffer, and membrane fractions were collected by ultracentrifugation. Membranes were solubilized in buffer containing n-dodecyl-β-D-maltoside (DDM) and cholesteryl hemisuccinate (CHS). Solubilized receptor–ligand complexes were purified by immobilized metal affinity chromatography on TALON resin, followed by gel filtration on a Superdex 200 Increase column (Supplementary Figure 1a, b). The purity of the purified H_1R_WT and $H_1R\_T112^{3.37}V$ used for ITC measurements were confirmed by SDS-PAGE. Fractions containing H_1R were pooled, concentrated, and stored at –80 °C until use.

HPLC analysis of doxepin E/Z isomers

Doxepin (E/Z mixture) was weighed and dissolved in the mobile phase consisting of 100 mM ammonium formate (pH 4.5) and acetonitrile (80:20, v/v) to a final concentration of 80 µM. Reverse-phase chromatography was performed using a Unison UK-C18 column (250 × 4.6 mm; Imtakt, Japan) on an HPLC system (JASCO, Japan). The mobile phase was delivered at a flow rate of 0.7 mL/min under a maximum pressure of 20 MPa and a column temperature of 37 °C. Detection was carried out at a wavelength of 254 nm, and chromatograms were analyzed using the ChromNAV Chromatography Data Station software (JASCO, Japan).

Isothermal Titration Calorimetry (ITC) Measurements

Purified H1R was placed in a Slide-A-Lyzer® Dialysis Cassette (MWCO 10,000; Thermo Fisher Scientific, USA) and dialyzed overnight against the measurement buffer containing 20 mM HEPES (pH 7.5), 150 mM NaCl, 10% glycerol, 0.025% n-dodecyl-β-D-maltoside (DDM), and 0.005% cholesteryl hemisuccinate (CHS). After dialysis, the sample was centrifuged at 16,000 × g for 10 min at 4 °C, and the supernatant was collected. The absorbance at 280 nm (A_{280}) and fluorescence intensity were measured, and assuming that approximately 80% of the total protein

corresponded to the target receptor, the samples were diluted with the dialysis buffer to final concentrations of 12 µM for H_1R_WT and 10 µM for H_1R_T112$^{3.37}$V).

Doxepin (E/Z mixture) was weighed and dissolved in water, and diluted in the measurement buffer at the concentration of 80 µM. Because the purchased amounts of E-doxepin and Z-doxepin (Toronto Research Chemicals Inc., Canada) were too small to be accurately weighed, the concentrations of the individual isomers were adjusted to match the A_{280} of the (E/Z) mixture solution. These solutions were used for the ITC binding experiments.

ITC measurements were performed using a MicroCal PEAQ-ITC instrument (Malvern Panalytical, UK). Milli-Q water was placed in the reference cell, while the sample cell contained the dialyzed H_1R (WT or T112$^{3.34}$V). The titration syringe was filled with doxepin solutions prepared in the same dialysis buffer. Measurements were conducted at 25 °C with the following parameters: spacing = 100 s, initial delay = 120 s, first injection = 0.4 µL, and injections 2–19 = 2 µL. For E- and Z-doxepin, ITC data were reanalyzed using the corrected ligand concentrations described above. All data analyses were performed using ORIGIN 7 (OriginLab, USA).

Correction of E- and Z-isomer concentrations based on absorption spectra and ITC stoichiometry

Absorption spectra of each doxepin solution were recorded between 220 and 320 nm, showing that the Z-isomer exhibited a higher absorbance in the shorter-wavelength region than the E-isomer (Supplementary Figure 2). Although the molar extinction coefficients of the E- and Z-isomers were not available, the monograph for doxepin hydrochloride in the U.S. Pharmacopeia (USP 31) indicates that HPLC analysis of doxepin isomers employs detection at 254 nm [3]. Therefore, it was assumed that the molar extinction coefficients of the E- and Z-isomers are approximately equal at 254 nm. The 80 µM (E/Z) mixture exhibited A_{254} = 0.652. From the absorption spectra, the A_{254} value of Z-isomer, which had been adjusted to match the A_{280} of the (E/Z) mixture, was approximately 1.28-fold higher than that of E-isomer—consistent with the stoichiometric ratio (1.25) determined by ITC. These findings support the assumption that the molar extinction coefficients of the E- and Z-isomers are nearly identical at 254 nm, and that the Z-isomer solution used in ITC measurements was approximately 1.28-fold more concentrated than the E-isomer solution. HPLC analysis at 254 nm indicated that the isomeric composition of the (E/Z) mixture was E:Z = 82:18. Based on these results, the effective concentrations of E- and Z-isomer used in the ITC experiments were recalculated to be 76 µM and 96 µM, respectively.

Cluster analysis of molecular dynamics simulations

Doxepin conformations obtained from our previous MD simulations of doxepin in solution and in complex with H_1R of WT and T112$^{3.37}$V mutant forms[1], were clustered using a *k*-means algorithm based on heavy atom RMSD [4]. Clustering was performed with the *clsuter* module in AMBER cpptraj program [5]. A total of 2,000 doxepin conformations were analyzed for each simulation condition.

References

1. Kaneko H, Korenaga R, Nakamura R, Kawai S, Ando T, Shiroishi M. Binding characteristics of the doxepin E/Z-isomers to the histamine H(1) receptor revealed by receptor-bound ligand analysis and molecular dynamics study. J Mol Recognit. 2024;37(5):e3098. doi:10.1002/jmr.3098.

2. Kota J, Gilstring CF, Ljungdahl PO. Membrane chaperone Shr3 assists in folding amino acid permeases preventing precocious ERAD. J Cell Biol. 2007;176(5):617-28. doi:10.1083/jcb.200612100.

3. Monograph: USP. Doxepin Hydrochloride. In: USP–NF. Rockville, MD: USP; Jun 1, 2018. https://www.uspnf.com/sites/default/files/usp_pdf/EN/USPNF/errata467DoxepinHydrochloride.pdf.

4. MacQueen J, editor. Some methods for classification and analysis of multivariate observations1967.

5. Case DA, Aktulga HM, Belfon K, Ben-Shalom IY, Berryman JT, Brozell SR et al. AMBER 2022. University of California, San Francisco2022.

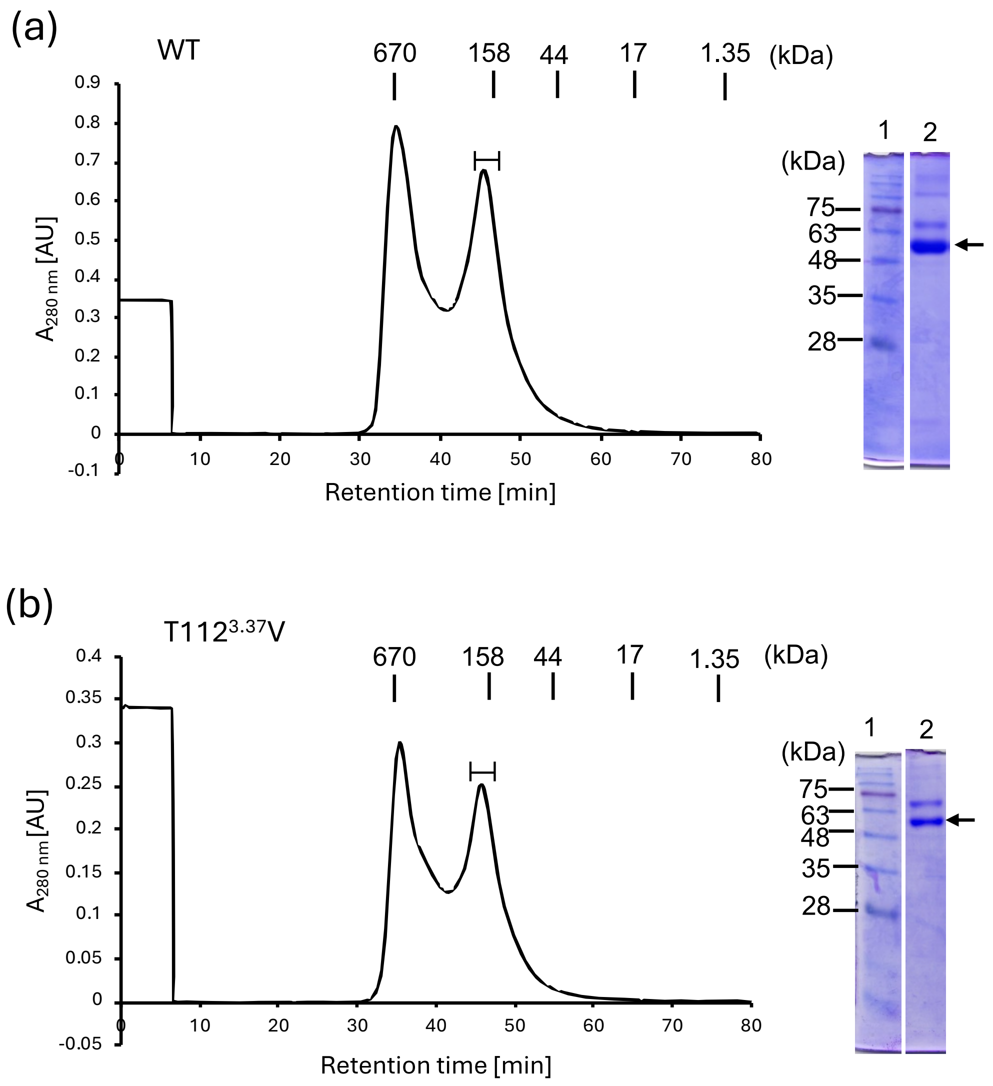

Supplementary Figure 1

Purification of H_1R_WT (a) and H_1R_T112$^{3.37}$V (b) by size-exclusion chromatography using a Superdex 200 Increase 10/300 column. Peak fractions within the range shown in the chromatogram were collected and used for ITC measurements. SDS-PAGE analysis of the purified protein is shown in the right panels. Lane 1, molecular weight marker; lane 2, purified H_1R. The arrow indicates the H_1R protein band.

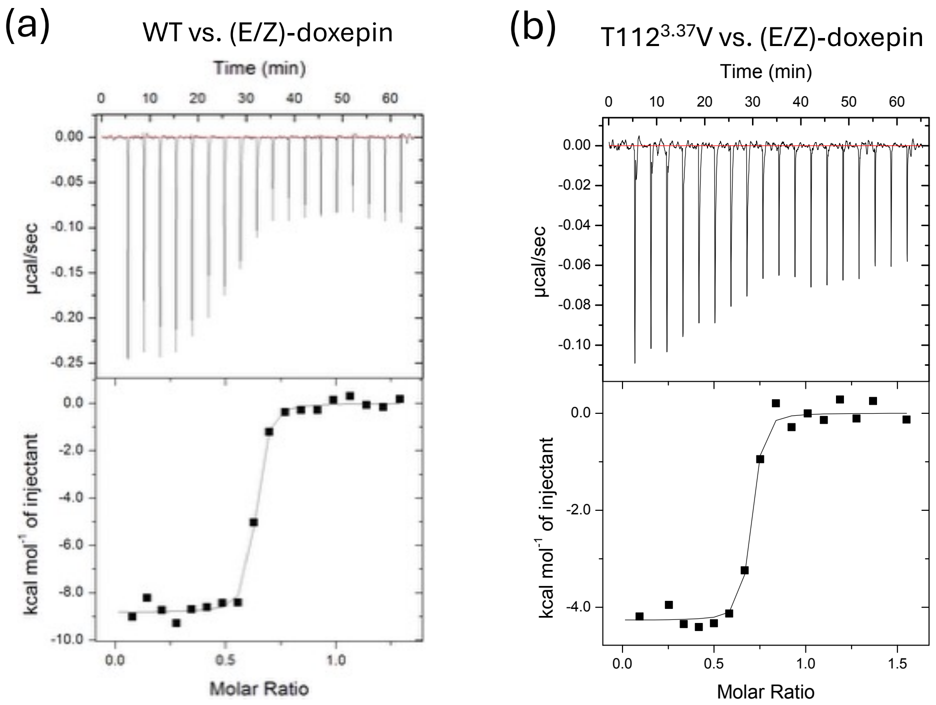

Supplementary Figure 2

ITC thermograms (upper panels) and binding isotherms (lower panels) for the interactions of doxepin (E/Z mixture) with (a) H_1R_WT and (b) H_1R_T112$^{3.37}$V.

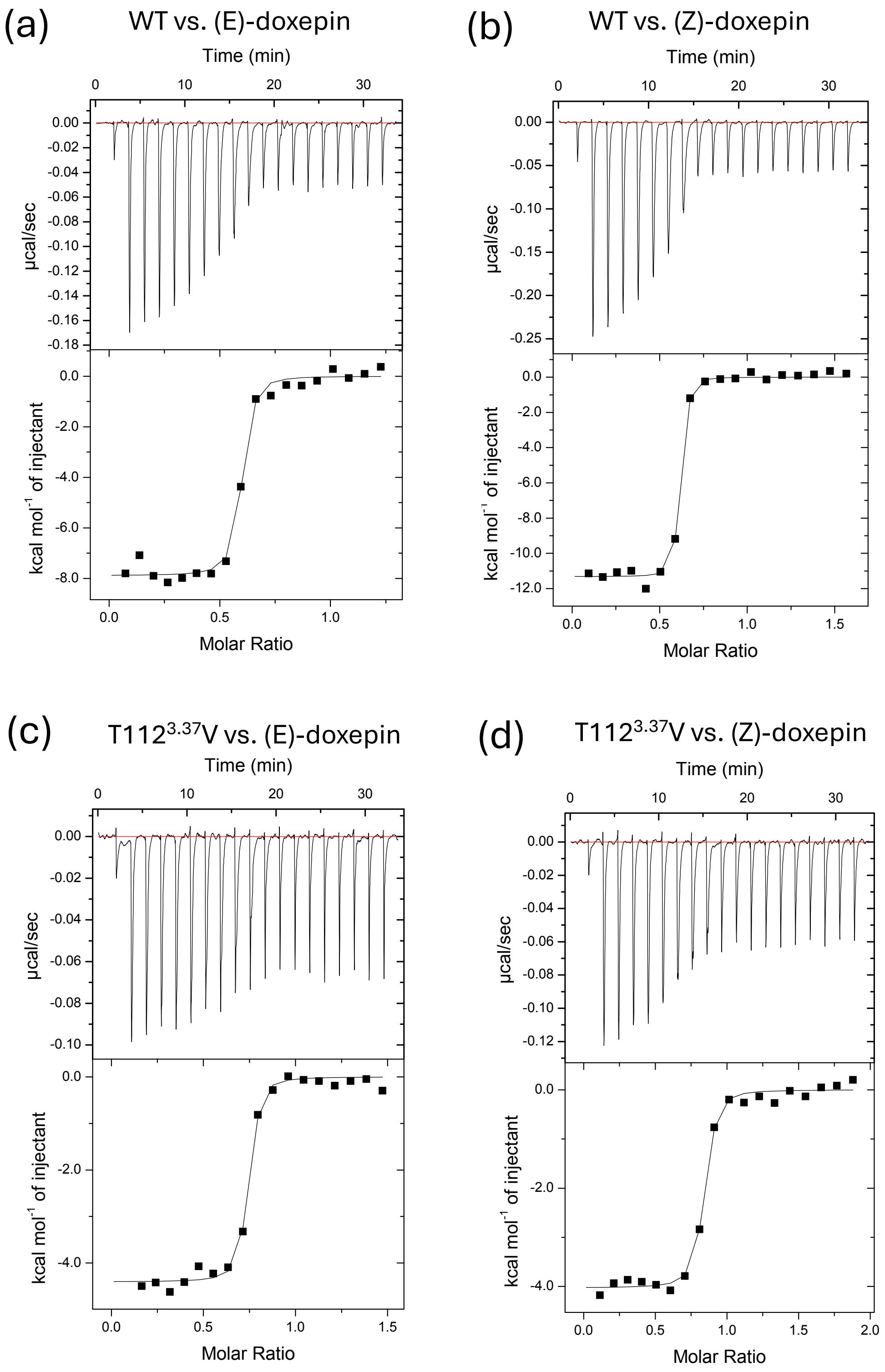

Supplementary Figure 3

(a, b) ITC thermograms (upper panels) and binding isotherms (lower panels) for the interactions between H_1R_WT and doxepin: (a) E-isomer and (b) Z-isomer.

(c, d) ITC thermograms (upper panels) and binding isotherms (lower panels) for the interactions between H_1R_T112$^{3.37}$V and doxepin: (c) E-isomer and (d) Z-isomer.

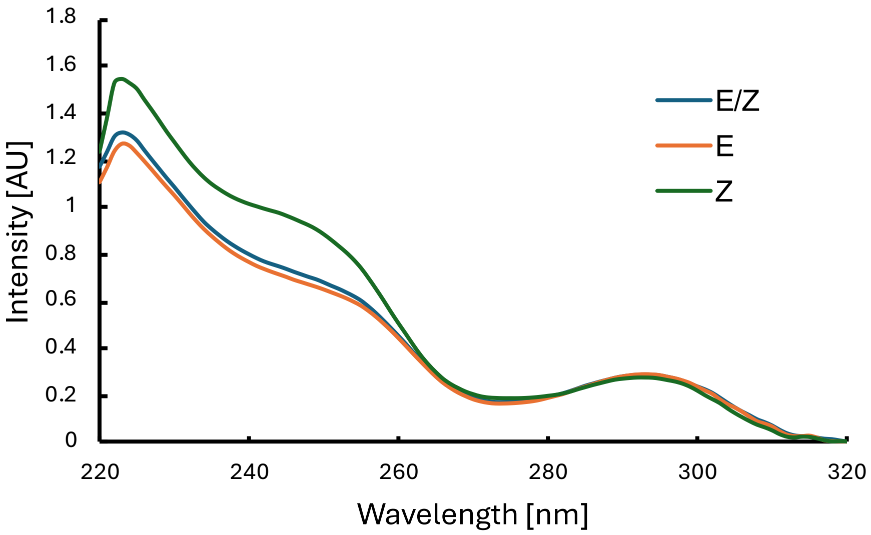

Supplementary Figure 4

UV–visible absorption spectra of doxepin (E/Z mixture), E-isomer, and Z-isomer in the range of 220–320 nm, with the concentrations adjusted to match the absorbance at 280 nm.
